# Influence of Increased Intra-Abdominal Pressure on the Validity of Ultrasound-Derived Inferior Vena Cava Measurements for Estimating Central Venous Pressure

**DOI:** 10.3390/jcm14113684

**Published:** 2025-05-24

**Authors:** Mia Rora Bertović, Vladimir Trkulja, Ela Ćurčić Karabaić, Sara Šundalić, Luka Bielen, Toni Ivičić, Radovan Radonić

**Affiliations:** 1 Department of Intensive Care Unit, Division of Internal Medicine, University Hospital Center Zagreb, 10000 Zagreb, Croatia; curcic.ela@gmail.com (E.Ć.K.); ssundalic@gmail.com (S.Š.); luka.bielen@yahoo.com (L.B.); toni.jivicin@gmail.com (T.I.); rradonic@gmail.com (R.R.); 2 Department of Internal Medicine, University of Zagreb School of Medicine, 10000 Zagreb, Croatia; 3 Department of Pharmacology, University of Zagreb School of Medicine, 10000 Zagreb, Croatia; vladimir.trkulja@mef.hr

**Keywords:** inferior vena cava diameter, ultrasound, central venous pressure, intra-abdominal pressure

## Abstract

**Background:** Ultrasound-based assessment of the inferior vena cava (IVC) is a widely used, non-invasive tool for estimating volume status and central venous pressure (CVP) in critically ill patients. However, elevated intra-abdominal pressure (IAP) may distort IVC measurements, reducing the accuracy of CVP estimation. This study aimed to quantify the effect of varying IAP on IVC diameters and evaluate the accuracy of ultrasound-based CVP predictions under such conditions. **Methods:** A prospective study was conducted including two groups of adult critically ill patients: one with spontaneously elevated IAP due to ascites (*n* = 36), undergoing stepwise pressure reduction via paracentesis, and one with normal baseline IAP (*n* = 30), undergoing stepwise pressure elevation using an abdominal belt with an inflatable balloon. End-inspiratory and end-expiratory IVC diameters and CVP were repeatedly measured at different IAP levels. Agreement between predicted and measured CVP was assessed using Gwet’s agreement coefficient, and a correction model for IVC diameters was developed based on IAP categories. **Results:** Increasing IAP led to a progressive reduction in both inspiratory and expiratory IVC diameters, while CVP showed no consistent trend. Predictive accuracy declined with rising IAP, with Gwet’s agreement coefficient decreasing from 0.851 (95 percent confidence interval: 0.750–0.952) at normal pressure to 0.392 (95 percent confidence interval: 0.141–0.642) at IAP above 25 mmHg. Applying the correction model improved prediction accuracy, with Gwet’s agreement coefficient increasing to 0.749 (95 percent confidence interval: 0.589–0.908) at the highest IAP category. **Conclusions:** Elevated IAP significantly alters IVC diameters and reduces the reliability of ultrasound-based CVP estimation. A correction model based on IAP improves predictive accuracy and may enhance volume assessment in critically ill patients. Further validation is warranted.

## 1. Introduction

Inferior vena cava (IVC) ultrasonography is widely adopted in managing fluid therapy in critically ill patients [1,2,3,4,5,6]. The IVC diameter, which varies with intravascular volume and the respiratory cycle, typically decreases during inspiration and increases during expiration due to changes in intrathoracic pressure [7,8]. This variation is quantified by the inferior vena cava collapsibility index (IVCCI), which tends to be high in hypovolemia and low in hypervolemia or elevated central venous pressure (CVP) [9,10,11]. Several simplified approaches for estimating CVP based on ultrasound-derived IVC parameters have been proposed to support rapid and informed decision-making in the critical care setting [12,13,14,15] (Appendix SA, Table S1).

Beyond the patient’s volume status, multiple physiological and technical factors may affect the reliability of IVC measurements and their interpretation. The extent of intrathoracic pressure changes during respiration can significantly influence IVC dynamics and the IVCCI [7,16]. In patients on mechanical ventilation or those with abnormal respiratory patterns (e.g., obstructive lung disease), inspiratory fluctuations may be exaggerated or absent, leading to inaccurate assessments. In addition, right heart dysfunction, such as right ventricular infarction, pulmonary embolism, cardiac tamponade, or severe tricuspid regurgitation, can alter venous return and cause IVC dilation with reduced collapsibility, which may not reflect actual CVP [17]. Additional factors such as patient body habitus, vascular tone, and imaging limitations (e.g., obesity or poor acoustic windows) further contribute to variability in measurements. Via et al. (2016) identified ten clinical scenarios in which IVC ultrasound may fail to accurately predict fluid responsiveness or volume status due to these and other interacting variables [18].

It is well known that elevated intra-abdominal pressure (IAP) reduces the diameter of the IVC, with higher pressures leading to a narrower diameter; however, this remains an under-researched area, with the limited available literature [19,20,21,22,23,24]. Normal IAP typically ranges from 5 to 7 mmHg (7 to 10 cm H_2_O), while intra-abdominal hypertension is defined as IAP exceeding 12 mmHg, classified into four grades: Grade I (IAP 12–15 mmHg), Grade II (IAP 16–20 mmHg), Grade III (IAP 21–25 mmHg), and Grade IV (IAP > 25 mmHg) [25,26]. Intra-abdominal hypertension is more prevalent in critically ill patients than previously recognized, affecting between 64% and 93% of patients in intensive care units [27,28]. Elevated IAP compromises organ system function; hence, its early recognition is important [29,30]. The most practical method for IAP estimation is intravesical pressure measurement [25,31].

The aim of this study was to establish a quantitative relationship between varying IAP levels, IVC diameter, and IVCCI to improve the accuracy of CVP estimation under conditions of elevated IAP.

## 2. Materials and Methods

### 2.1. Study Outline

This prospective study enrolled consecutive adult patients managed at the Intensive Care Unit (ICU), University Hospital Center Zagreb, Zagreb, Croatia, between 15 September 2020 and 1 November 2023. This study was approved by the Institutional Ethics Committee (approval class: 8.1-18/183-2, number 02/21 AG, date of approval: 30 September 2018). Written informed consent was obtained from all patients or their caregivers for participation in this study and for the use of anonymized data for research purposes and scientific publication. Eligible patients were those with both a central venous catheter (to enable measurement of the CVP) and a urinary catheter in place (to enable IAP measurement) due to their medical condition. Two groups of patients were included: (i) patients with spontaneously increased IAP due to ascites requiring removal, as indicated by the attending physician, to study the effects of progressively declining IAP induced by gradual ascites evacuation on simultaneously measured CVP and IVC diameters; and (ii) patients with normal IAP, to study the effects of progressively increasing IAP induced by a wide abdominal belt and an inflatable balloon. All measurements were performed by two trained and experienced intensive care specialists. The IVC diameters (end-inspiratory and end-expiratory) were used to calculate the IVCCI. Agreement between the measured CVP and the predicted CVP, based on IVC diameters and IVCCI (see Appendix SA, Table S1), was assessed at different levels of IAP. Based on the differences between the measured IVC diameters at “normal” IAP and those at increased IAP levels, a correction for IVC diameters under elevated IAP conditions was developed. Finally, to evaluate whether this correction improved the accuracy of CVP predictions, the agreement between the predicted and measured CVP at elevated IAP levels was compared between predictions using the correction and those without it.

### 2.2. Patients

Inclusion criteria were age ≥ 18 years, the presence of a central venous catheter and a urinary catheter placed for reasons unrelated to this study, and informed consent. Additional inclusion criteria for the group with spontaneously increased IAP included elevated IAP due to tension ascites requiring removal. Additional inclusion criteria for the second group included normal IAP. Exclusion criteria were pregnancy, cardiac tamponade, tricuspid regurgitation, right-sided heart failure, obstructive pulmonary diseases, thrombosis or tumor infiltration of the IVC, amputated leg, intravascular devices inserted into the IVC, mechanically ventilated patients unable to trigger ventilatory support, positive end-expiratory pressure > 5 cm H_2_O or pressure support > 12 cm H_2_O in mechanically ventilated patients, spontaneously increased IAP due to causes other than ascites, and inadequate IVC visualization. Additional exclusion criteria for a second group with normal IAP included obesity (BMI > 30 kg/m^2^ or abdominal circumference > 120 cm) and conditions where artificially increasing IAP could cause harm or discomfort. These conditions included intra-abdominal inflammatory processes, abdominal wall injury or inflammation, aortic aneurysm, and abdominal or thoracic surgery within two months prior to this study.

### 2.3. Ultrasound Techniques for Measuring the Inferior Vena Cava

Using an epigastric approach or, alternatively, an intercostal approach with the liver as an acoustic window (no more lateral than the midclavicular line), the IVC was visualized in both transverse and longitudinal cross-sections. The maximal anteroposterior diameter of the transverse IVC cross-section was measured first. A longitudinal view was then obtained by tilting the probe to identify the IVC image with the widest diameter, aiming to match the maximal diameter recorded in the transverse view. All measurements followed the guidelines of the American Society of Echocardiography [12] and were performed proximal to the junction of the hepatic veins, approximately 0.5 to 3 cm from the ostium of the right atrium. Diameter measurements were taken from B-mode video clips, where diaphragm movements allowed for accurate identification of end-expiration and end-inspiration, corresponding to the widest and narrowest IVC diameters during the respiratory cycle. Ultrasound imaging was performed using a GE Logiq 8 device with a convex C1–5 MHz probe. The IVCCI was calculated as (widest diameter − narrowest diameter)/widest diameter. Before any change in IAP, baseline measurements of IVC diameter at end-inspiration and end-expiration were obtained, capturing the narrowest and widest diameters. In the group with spontaneously increased IAP, measurements were repeated at each step of IAP reduction by 1 mmHg. In the second group with normal IAP, measurements were repeated at IAP elevations of 5, 10, and 15 mmHg above baseline.

### 2.4. Measurement of Intra-Abdominal Pressure

The patient is positioned supine. The urinary catheter is connected to an infusion set attached to a measurement scale filled with sterile saline solution. A T-connector allows for saline infusion into the bladder. The bladder is slowly filled with 25 mL of sterile saline to provide adequate fluid for pressure transmission without causing bladder distension, which could affect the pressure level. The measurement scale is calibrated and aligned so that zero corresponds to the level of the patient’s mid-axillary line, the standard reference point for abdominal pressure measurement. Once the system is prepared, the T-connector is adjusted to fill the scale according to the principle of communicating vessels. IAP is measured at the end of expiration to ensure a baseline reading is unaffected by additional diaphragmatic influences.

### 2.5. Measurement of Central Venous Pressure

To measure CVP using a central venous catheter and a scale calibrated in millimeters of mercury, the patient is positioned supine with the head of the bed in a horizontal position. The standard reference point for measurement is the level of the right atrium, aligned with the mid-axillary line, which is considered the zero point for CVP. The system is filled with a normal saline solution, allowing it to flow into the venous system until the pressure equilibrates and the flow stops. The CVP reading is taken at end-expiration to capture the most accurate baseline value. Before any changes in IAP, the first CVP measurement was performed. In the group with spontaneously elevated IAP, all measurements were repeated at each step of IAP reduction by 1 mmHg. In the second group with normal IAP, all measurements were repeated at IAP elevations of 5, 10, and 15 mmHg above the baseline value.

### 2.6. Ultrasound-Guided Ascites Removal

Paracentesis was performed at the standard site, located at the lateral third of the line connecting the umbilicus and the left anterior superior iliac spine. Ultrasound was used to confirm the suitability of the puncture site. An 18-gauge needle attached to a drainage tube was used for ascites drainage, with the tube connected to a measuring device to quantify fluid output. Ascitic fluid was gradually evacuated until the entire removable volume was drained.

### 2.7. Artificial Elevation of Intra-Abdominal Pressure

IAP was artificially increased by applying a wide belt (SAM Pelvic Sling II, SAM Medical Products, Wilsonville, OR, USA) around the abdomen. Under the belt, a standard inflatable balloon from a medical blood pressure cuff was used and gradually inflated with air to increase IAP by 5, 10, and 15 mmHg above baseline levels.

### 2.8. Predicting Central Venous Pressure

All measured CVP values were classified into four categories: 0–5 mmHg, 6–10 mmHg, 11–15 mmHg, and 16–20 mmHg. None of the observed values exceeded 20 mmHg. Predicted category classifications were generated based on the widest IVC diameter and the IVCCI (Appendix SA, Table S1).

### 2.9. Correction of the Measured Inferior Vena Cava Diameters

All measured IAP values were grouped into five categories: 4–10 mmHg (considered “normal” and used as the reference) and four categories corresponding to Grades I–IV of intra-abdominal hypertension: 11–15, 16–20, 21–25, and >25 mmHg [23,25]. To quantify the influence of elevated IAP on IVC diameters, linear models were fitted for both end-inspiratory and end-expiratory IVC measurements, adjusting for age, sex, and mechanical ventilation status. These models were used to estimate the difference in IVC diameters between each increased IAP category and the reference. The resulting point estimates were then applied to correct measured IVC diameters: for values within the midpoint of an IAP category, the corresponding point estimate was added; for values near the upper or lower bounds, the upper and lower limits of the 95% confidence intervals (CI) were used, respectively. Full details of the correction procedure are presented in Appendix SB.

### 2.10. Data Analysis

Data were analyzed separately in patients with spontaneously increased IAP, patients with artificially increased IAP, and in the combined cohorts. In patients with spontaneously increased IAP, data showed the following: (i) effect of declining IAP (by 1 mmHg change) on CVP, end-inspiratory and end-expiratory IVC diameters, and IVCCI, estimated with adjustment for age, use of mechanical ventilation, sex and baseline value of the outcome variable by fitting random intercept and slope mixed models (REML estimation, Kenward–Roger degrees of freedom); (ii) agreement between the measured and predicted CVP (4 categories) at baseline, after 5 ascites removals, and after complete removal of ascites. In patients with artificially increased IAP, data showed the following: (i) effect of IAP increase (by 5, 10, or 15 mmHg) on CVP, IVC diameters, and IVCCI estimated by fitting mixed models with adjustment for age, sex, mechanical ventilation, and baseline value of the outcome variable; (ii) agreement between the measured and predicted CVP at baseline, and at 5, 10 and 15 mmHg increase vs. baseline. In the combined patient cohorts, data showed the following: (i) estimation of differences in measured end-inspiratory and end-expiratory IVC diameters between the 4 categories of increased IAP and the reference category (IAP 4–10 mmHg) by fitting mixed models with adjustment for age, sex, and use of mechanical ventilation; (ii) correction of IVC diameters measured at increased IAP levels based on the estimated differences (Appendix SB); (iii) comparison of agreement between predicted and actual CVP categories between predictions that used the correction and those that did not. The mixed models were fitted in SAS 9.4 for Windows (SAS Inc., Cary, NC, USA). In the agreement analysis, we determined the weighted chance-corrected Gwet’s agreement coefficient (AC2) by using linear weights in line with the view that the distance between each of the four levels of CVP was equally relevant (package irr.CAC [32] (pp. 73–100) in R [33]).

## 3. Results

### 3.1. Patients

We enrolled a total of 66 patients (median age 63 years, 69.7% men, 42.4% mechanically ventilated), 36 with spontaneously increased IAP with ascites (median age 58 years, 80.6% men, 50% mechanically ventilated) almost exclusively suffering from liver cirrhosis, and 30 with “normal” IAP in whom it was artificially increased (median age 72 years, 56.7% men, 33.3% mechanically ventilated) (Table 1). Baseline IAP (overall median 15.5 mmHg) and CVP (overall median 11.5 mmHg) were higher in the former than in the latter subset (22.0 vs. 9 mmHg, and 14.5 vs. 9 mmHg respectively) (Table 1), whereas baseline end-expiratory IVC diameter (overall median 1.5 cm) and collapsibility index (overall median 30.6%) were lower (1.1 vs. 1.9 cm, and 7.4% vs. 48.1%, respectively) (Table 1), with similar end-inspiratory IVC diameter (0.9 vs. 1.1 cm, overall 1.0 cm) (Table 1).

### 3.2. Relationship Between Decreasing Intra-Abdominal Pressure and Hemodynamic Indicators

In patients with spontaneously increased IAP with ascites, IAP linearly decreased across the increasing number of ascite withdrawals (Appendix SC, Figure S1). A minimum of 3 and a maximum of 13 post-baseline measurements were taken, with an IAP reduction from a median of 22 at baseline to a median of 13 mmHg after complete evacuation (Appendix SC, Table S2).

**Figure 1:**
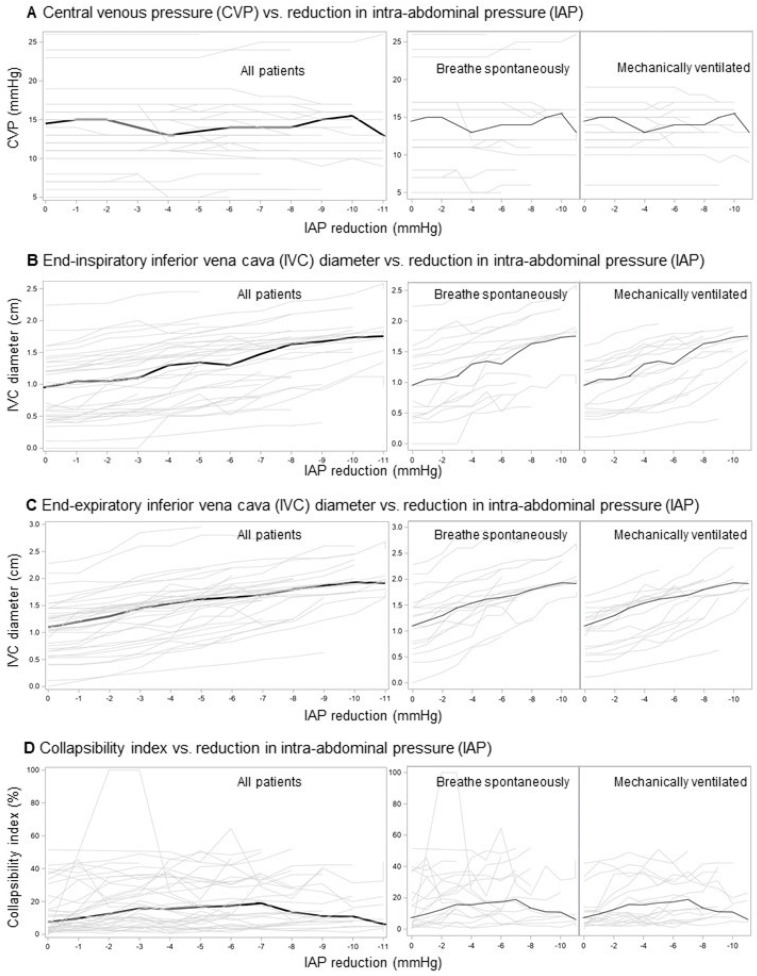
Relationship between reduction in intra-abdominal pressure (IAP) (induced by ascites removal) and hemodynamic indicators in patients with spontaneously increased IAP and ascites—raw data. Central venous pressure (CVP) (**A**), end-inspiratory (**B**), end-expiratory (**C**), inferior vena cava diameters (IVC), and inferior vena cava collapsibility index (IVCCI) (**D**). Thin gray lines—individual data; thick black lines—median.

CVP showed no consistent trend of change with declining IAP (Figure 1A), whereas the end-inspiratory (Figure 1B) and end-expiratory IVC (Figure 1C) diameters progressively increased: 1 mmHg IAP reduction resulted in an (adjusted) increase of 0.07 cm (95% CI 0.06–0.08) and 0.10 cm (95% CI 0.09–0.12), respectively. The IVCCI showed a quadratic relationship with the declining IAP (Figure 1D): 1 mmHg reduction resulted in an initial (adjusted) increase of 6.6% (95% CI 2.7–10.5) (up to a reduction of around 6 mmHg), followed by a decrease of 1.1% (95% CI 0.6–2.2).

**Figure 2:**
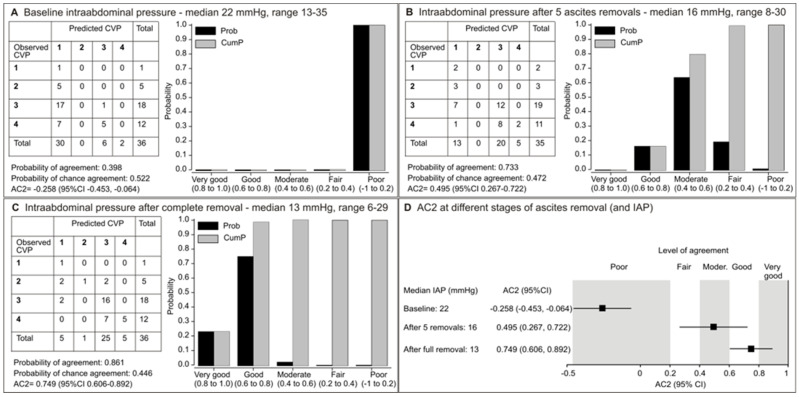
Agreement between the predicted and measured central venous pressure (CVP mmHg) classified into 4 categories: [0–5 (1), 6–10 (2), 11–15 (3), 16–20 (4)] in patients with spontaneously increased intra-abdominal pressure (IAP) at different stages of ascites removal and IAP values (**A**–**C**), and evolution of the level of agreement (**D**). Shown are probabilities (P), cumulative probabilities (CumP), Gwet’s AC2 coefficient, and Altman’s benchmark values for AC2 (poor–very good).

Before the ascites removal (baseline), there was no agreement between the predicted and actual CVP (Figure 2A). After five ascites removals, the probability of good or moderate agreement was cumulatively 80%, and AC2 (0.495, 95% CI 0.267–0.722) was in the range of moderate agreement (Figure 2B). After complete ascites removal, the cumulative probability of very good or good agreement was 98%, and AC2 (0.749, 95% CI 0.606–0.892) was in the range of good agreement (Figure 2C). With decreasing IAP, the agreement between the predicted and actual CVP improved from no agreement to good agreement (Figure 2D).

### 3.3. Relationship Between Increasing Intra-Abdominal Pressure and Hemodynamics Indicators

**Figure 3:**
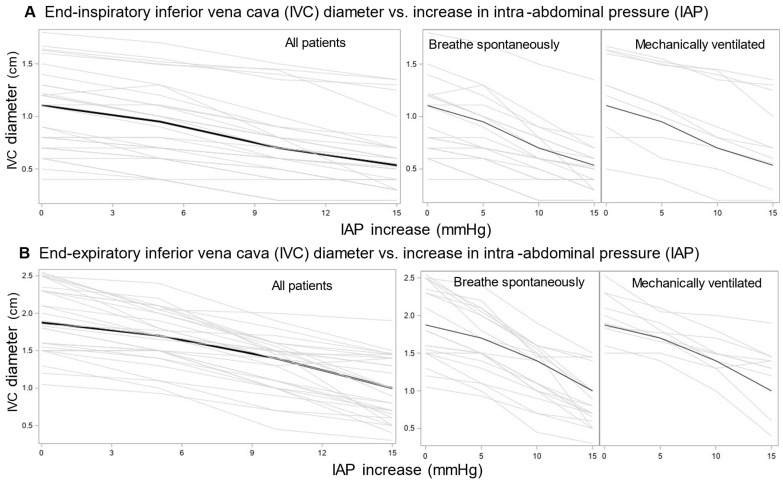
Relationship between induced (by belt) increase in intra-abdominal pressure (IAP) and hemodynamic indicators in patients with normal IAP at baseline—raw data. End-inspiratory (**A**) and end-expiratory (**B**) inferior vena cava diameters (IVC). Thin gray lines—individual data; thick black lines—median.

Induction of IAP in patients with normal IAP at baseline resulted in median IAP values of 14.0, 19.0, and 24 mmHg, respectively (Appendix SD, Table S3). Central venous pressure (CVP) and IVCCI showed no consistent change trend with increasing IAP (Appendix SD, Figure S2), whereas the end-inspiratory and end-expiratory IVC diameters progressively decreased (Figure 3): IAP increases of 5, 10 and 15 mmHg above baseline value resulted in (adjusted) reductions of end-inspiratory IVC diameter of −0.12 (95% CI –0.18, −0.07), −0.31 (−0.36, −0.26), and −0.46 (−0.51, −0.40) cm, respectively, and of end-expiratory IVC diameter of −0.26 (−0.35, −0.16), −0.61 (−0.71, −0.52), and −0.94 (−1.03, −0.84) cm, respectively.

**Figure 4:**
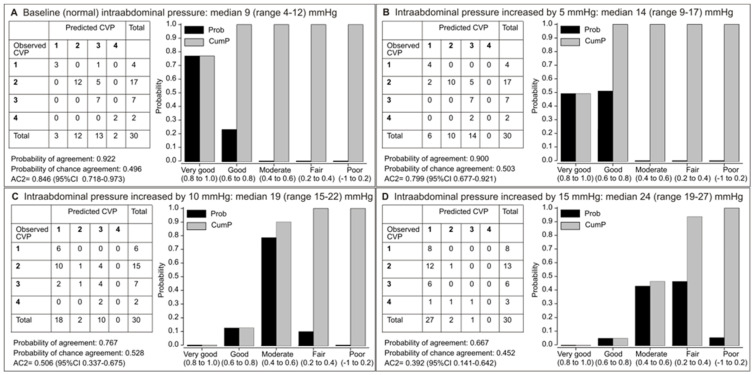
Agreement between the predicted and measured central venous pressure (CVP mmHg) classified into 4 categories: [0–5 (1), 6–10 (2), 11–15 (3), 16–20 (4)] in patients with normal intra-abdominal pressure (IAP) at baseline, at different stages of artificially increased IAP (baseline—(**A**), increased by 5 mmHg—(**B**), increased by 10 mmHg—(**C**), increased by 15 mmHg—(**D**)). Shown are probabilities (P), cumulative probabilities (CumP), and Gwet’s AC2 coefficient.

At baseline (normal IAP), the probability of very good (77%) and good (22%) agreement was cumulatively 99%, and AC2 (0.846, 95% CI 0.718–0.973) was in the range of very good (Figure 4A). At IAP increased by 5 mmHg, the probability of very good agreement decreased; the probability of good agreement increased, and their cumulative probability was still 98%, but AC2 (0.799, 95% CI 0.677–0.921) was in the range of good-to-very good (Figure 4B). With IAP increases of 10 mmHg (Figure 4C) and 15 mmHg (Figure 4D), probabilities of agreement shifted to predominantly moderate or moderate-to-fair, and AC2 values (0.506, 95% CI 0.337–0.675; 0.392, 95% CI 0.141–0.642) were in the ranges of moderate and fair, respectively (Figure 4C,D).

**Figure 5:**
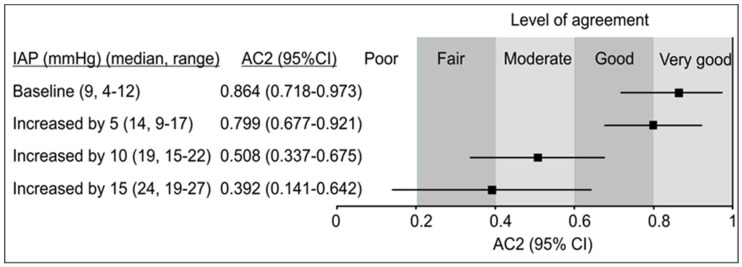
Summary of agreement analysis between the predicted and observed central venous pressure (CVP) at different levels of induced increase in intra-abdominal pressure (IAP). Shown is Gwet’s AC2 coefficient with Altman’s benchmark values (very-good-to-poor).

Hence, when intra-abdominal pressure (IAP) increased by 5–15 mmHg above baseline, the level of agreement decreased from very good to only fair (Figure 5).

### 3.4. Corrected Inferior Vena Cava Diameters

To evaluate the extent to which IAP affected the measured IVC diameters and to determine the potential benefit of a correction approach, all available data from both patient groups (*n* = 454 observations) were analyzed. The data were grouped into five IAP categories: reference (4–10 mmHg) and four elevated categories (11–15, 16–20, 21–25, and 26–35 mmHg). For each IAP category, adjusted mean end-inspiratory and end-expiratory IVC diameters were estimated and compared with the reference category (Appendix SE, Tables S4 and S5). The differences obtained were used to calculate correction values for each IAP category.

Correction values were then added to the measured IVC diameters to compensate for IAP-induced narrowing. For each IAP category, specific correction increments were defined for both inspiratory and expiratory diameters, as presented in Table 2. For example, at an IAP of 24–25 mmHg, the correction values were +0.63 cm (inspiration) and +1.02 cm (expiration). These adjustments aimed to restore IVC diameters to levels equivalent to those expected under normal IAP.

**Figure 6:**
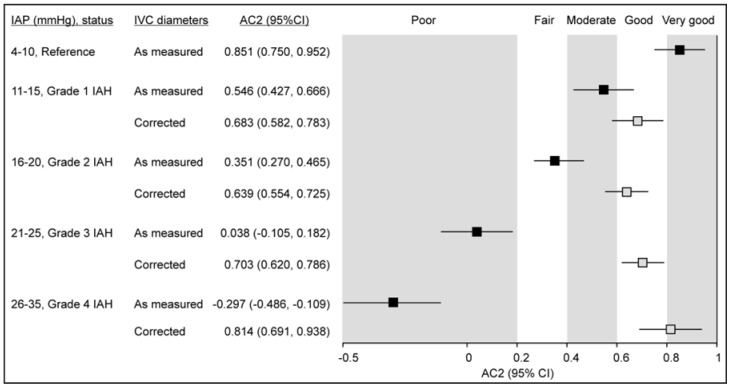
Agreement (Gwet’s AC2 coefficient) between measured central venous pressure (CVP) and predictions based on inferior vena cava (IVC) diameters measured at different levels of intra-abdominal pressure (IAP)—either normal (reference) or elevated, corresponding to Grades 1–4 of intra-abdominal hypertension. Comparisons are shown for diameters measured versus those after applying the correction model. Agreement levels are interpreted according to Altman’s benchmark categories (very-good-to-poor).

After applying the correction, the agreement between predicted and observed CVP categories significantly improved across all IAP levels. As shown in Figure 6, uncorrected diameters were associated with a progressive decline in predictive accuracy, with AC2 decreasing to 0.392 (95% CI 0.141–0.642) at IAP > 25 mmHg. In contrast, the use of corrected diameters improved classification performance, with AC2 reaching 0.749 (95% CI 0.589–0.908) in the same IAP range. These results support the utility of IVC diameter correction in enhancing non-invasive estimation of CVP in patients with elevated IAP.

## 4. Discussion

An accurate understanding of hemodynamic status in critically ill patients is essential for effective volume management. The use of IVC ultrasound, a readily available and non-invasive tool, for this purpose, has garnered much attention [34,35,36,37,38]. Among the multifactorial influences on the diameter of the IVC in assessing volume status, the impact of elevated IAP appears to be the least studied. In the present study, we specifically focused on assessing the effects of increased IAP on ultrasound-determined IVC diameters and the validity of CVP predictions based on IVC diameters measured under conditions of increased IAP.

Previous studies addressing the relationship between IAP and IVC dynamics have been limited in several key ways [19,20,21,23,24]. Most involved small sample sizes lacked a full spectrum of IAP values and failed to apply stepwise pressure control or real-time dynamic ultrasonography. Many relied on static imaging modalities such as computed tomography or magnetic resonance imaging, which are limited by their inability to capture dynamic respiratory changes [19,20,24]. In particular, existing ultrasound-based studies rarely quantified the relationship between graded IAP levels and changes in IVC diameter or IVCCI [21,23]. Furthermore, prior work did not validate the accuracy of CVP estimation under elevated IAP conditions, nor did they propose correction methods to adjust for pressure-induced IVC distortion. These methodological shortcomings underscore the need for a more systematic and physiologically grounded investigation into this relationship.

For our research, we utilized the modified model of induced intra-abdominal hypertension described by Cavaliere et al. [21], which allowed for adjustable pressure levels and objective IAP measurements. This model was applied to a group of patients with normal IAP, where pressure was artificially and incrementally increased. Another group consisted of patients with elevated IAP due to ascites, providing an opportunity to monitor changes in IAP and IVC diameter as IAP gradually decreased with ascitic drainage. Most patients with ascites had liver cirrhosis, a condition commonly associated with specific pathophysiological derangements that could introduce systemic bias. In contrast, the group with artificially elevated IAP more closely represents the typical intensive care unit population; however, systemic error may arise as this approach can differ from the actual pathophysiological conditions of intra-abdominal hypertension.

The inclusion of both models allowed for cross-validation of observations between the effects of decreasing and increasing IAP, providing a broad spectrum of finely graded IAP values to enable a detailed assessment of the ‘dose–effect’ relationship. Both models yielded comparable results, supporting the validity of the artificially induced IAP model. The system combining the widest IVC diameter values with the IVCCI to predict CVP categories (Appendix SA, Table S1) demonstrated considerable accuracy under conditions of low or normal IAP. This was evidenced by very good agreement between predicted and observed CVP categories (Gwet’s agreement coefficient = 0.851, 95% confidence interval 0.750–0.952). Increasing IAP induced a linear decrease in both end-inspiratory and end-expiratory IVC diameters while decreasing IAP had the reverse effect. However, changes in IAP did not have a consistent effect on IVCCI. As expected, CVP remained unaffected by changes in IAP.

At higher IAP levels consistent with Grade 1–4 intra-abdominal hypertension, there was a progressive decline in the accuracy of CVP predictions, with agreement shifting from very good at normal IAP to poor at Grade 4. Notably, applying the correction model substantially restored prediction accuracy. The use of corrected IVC diameters improved classification performance at all IAP levels, with Gwet’s agreement coefficient reaching 0.749 (95% confidence interval 0.589–0.908) at IAP > 25 mmHg.

Although the corrections applied in this study may not be universally applicable, they demonstrate that the diagnostic accuracy of ultrasound-based CVP assessment can be improved when IAP is elevated. This study demonstrated solid internal validity. All measurements were conducted jointly by two experienced intensive care specialists, ensuring mutual supervision and adherence to standardized methods. The methods used to estimate IAP and measure CVP are well-established and valid [25,39,40] (p. 645). Considering CVP in terms of categories—hypovolemia (0–5 mmHg), normovolemia (6–10 mmHg), mild hypervolemia (11–15 mmHg), and hypervolemia (16–20 mmHg)—is clinically justified and informative. While the sample size was moderate, it was larger than in similar studies on this topic [21], enabling reasonable precision in critically ill patients.

This study’s exclusion criteria limited its external validity. Patients with increased IAP from causes other than ascites, those with conditions that might compromise respiration (e.g., chronic obstructive pulmonary disease, positive end-expiratory pressure > 5 cm H_2_O or pressure support > 12 cm H_2_O), conditions affecting ultrasound-based CVP assessment (e.g., tricuspid regurgitation, right-sided heart failure, intravascular devices in the IVC), and technical obstacles (e.g., obesity, inadequate IVC visualization) were excluded. While necessary to achieve this study’s objectives, these exclusions restricted this study to a subset of patients. Thus, this study should be regarded as demonstrating a reasonable concept that warrants further evaluation in larger, multicentric studies reflecting the complexity of the ICU population. The effects of IAP on IVC diameters were similar in spontaneously breathing patients and those on mechanical ventilation within the specified mechanical support limits. This consistency suggests comparable respiratory dynamics between the groups.

## 5. Conclusions

In critically ill patients with normal IAP, ultrasound-based assessment of IVC diameters enables accurate estimation of CVP, with agreement between predicted and measured CVP reaching very good levels (Gwet’s agreement coefficient = 0.851; 95% confidence interval 0.750–0.952). However, as IAP increases, both end-inspiratory and end-expiratory IVC diameters progressively decrease, leading to a substantial decline in predictive accuracy (Gwet’s agreement coefficient = 0.392; 95% confidence interval 0.141–0.642 at IAP > 25 mmHg). We developed a correction model based on IAP-related reductions in IVC diameter, which restored agreement between predicted and actual CVP values to good or very good levels even at the highest observed IAP category (IAP > 25 mmHg; Gwet’s agreement coefficient = 0.749, 95% confidence interval 0.589–0.908). While these findings suggest that IVC-based CVP estimates can be adjusted according to IAP, this model should currently be regarded as a proof-of-concept. It provides a foundation for future research aimed at validating and refining this approach before its integration into routine clinical use.

## Figures and Tables

**Table 1:** Patient characteristics: overall, for patients with spontaneously increased intra-abdominal pressure (IAP) and ascites (“Spontaneous”), and patients with artificially increased IAP (“Artificial”). Data are count (percent) or median (Q1, Q3; min, max).

	All Patients	Spontaneous	Artificial
*N*	66	36	30
Age (years)	63 (54–72; 21–86)	58 (47–66; 34–78)	72 (61–76; 21–86)
Men	46 (69.7)	29 (80.6)	17 (56.7)
Breathe spontaneously	38 (57.6)	18 (50)	20 (66.7)
Mechanically ventilated	28 (42.4)	18 (50)	10 (33.3)
BIPAP	16 (24.2)	11 (30.5)	5 (16.6)
ASB	11 (16.7)	6 (16.6)	5 (16.6)
SIMV	1 (1.5)	1 (2.8)	0
Sedated (midazolam)	8 (12.1)	7 (19.4)	1 (3.3)
Diagnosis			
Liver cirrhosis	32 (48.5)	30 (83.3)	2 (6.7)
Sepsis	13 (19.7)	1 (2.8)	12 (40.0)
Pneumonia	6 (9.1)	0	6 (20.0)
Cardiorespiratory arrest	3 (4.5)	0	3 (10.0)
Other	12 (18.2)	5 (13.9)	7 (23.3)
Baseline IAP (mmHg)	15.5 (9.8–22.2; 4–35)	22.0 (19–25; 13–25)	9.0 (7.7–10; 4.0–12.0)
Baseline CVP (mmHg)	11.5 (8–15; 4–20)	14.5 (11–16; 5–20)	9.0 (7.7–10; 4.0–12.0)
Baseline end-inspiratory IVC (cm)	1.0 (0.6–1.3; 0–2.2)	0.9 (0.5–1.3; 0–2.2)	1.1 (0.7–1.3; 0.4–1.8)
Baseline end-expiratory IVC (cm)	1.5 (1.0–1.9; 0–2.5)	1.1 (0.7–1.5; 0–2.3)	1.9 (1.5–2.3; 1.1–2.6)
Baseline collapsibility index (%)	30.6 (6.3–48.4; 0–68.8)	7.4 (2.7–22.4; 0–51.5)	48.1 (35.4–55.2; 8.7–68.7)

BIPAP—Bi-level Positive Airway Pressure; ASB—Assisted Spontaneous Breathing; SIMV—Synchronized Intermittent Mandatory Ventilation; IAP—intra-abdominal pressure; CVP—central venous pressure; IVC—inferior vena cava.

**Table 2:** Correction values (cm) to be added to measured inferior vena cava (IVC) diameters at different intra-abdominal (IAP) levels.

IAP (mmHg)	End-Inspiratory IVC	End-Expiratory IVC
4–10	none	none
11–12	+0.1	+0.24
13	+0.15	+0.31
14–15	+0.2	+0.39
16–17	+0.32	+0.58
18	+0.38	+0.65
19–20	+0.42	+0.72
21–22	+0.51	+0.86
23	+0.57	+0.94
24–25	+0.63	+1.02
26–28	+0.73	+1.21
29–31	+0.82	+1.33
32–35	+0.90	+1.44

IAP—intra-abdominal pressure; IVC—inferior vena cava.

## Data Availability

The raw data supporting the aggregation and conclusions of this article will be made available by the author upon request, subject to additional approval from the ethics committee.

## Supplementary Materials

The following supporting information can be downloaded at: https://www.mdpi.com/article/10.3390/jcm14113684/s1, Appendix SA: Selected guides to predict central venous pressure based on inferior vena cava diameters and the inferior vena cava collapsibility index; Table S1: Relationship between the widest inferior vena cava (IVC) diameter, inferior vena cava collapsibility index (IVCCI), and corresponding (estimated) central venous pressure (CVP); Appendix SB: Methods to correct inferior vena cava diameters measured at increased levels of the intra-abdominal pressure; Appendix SC: Patients with spontaneously increased intra-abdominal pressure and ascites: descriptive hemodynamics data; Table S2: Hemodynamics indicators; Figure S1: Development of intra-abdominal pressure (IAP) across the number of ascites withdrawalas; Appendix SD: Patients with normal intra-abdominal pressure at baseline and intra-abdominal pressure induction; Table S3: Effect of implementation of abdominal belt on intra-abdominal pressure (IAP) in patients with normal baseline IAP; Figure S2: Effect of increasing intra-abdominal pressure (IAP) on central venous pressure (CVP) (A) and the inferior vena cava collapsibility index (IVCCI) (B); Appendix SE: Correction of inferior vena cava diameters measured at increased intra-abdominal pressure; Table S4: Data used to estimate difference in measured end-inspiratory and end-expiratory inferior vena cava (IVC) diameters between those taken with intra-abdominal pressure (IAP) in the range between 4 and 10 mmHg (reference range), and those taken at higher values of IAP (11–15, 16–20, 21–25 or 26–35 mmHg); Table S5: Adjusted mean differences (95%CI) between the end-inspiratory or end-expiratory inferior vena cava diameters taken at normal intra-abdominal pressure (4–10 mmHg, reference), and at higher levels of IAP estimated based on data in Table S1.

## Abbreviations

The following abbreviations are used in this manuscript:

CI	confidence interval
AC2	agreement coefficient
CVP	central venous pressure
ICU	intensive care unit
IAP	intra-abdominal pressure
IVC	inferior vena cava
IVCCI	inferior vena cava collapsibility index
