# Supplementary material for: Influence of Increased Intra-Abdominal Pressure on the Validity of Ultrasound-Derived Inferior Vena Cava Measurements for Estimating Central Venous Pressure
Source: J Clin Med. 2025 May 24;14(11):3684. doi: 10.3390/jcm14113684 (PMC12156460; doi:10.3390/jcm14113684)
Supplement: Supplementary file 1 [file jcm-14-03684-s001.zip › jcm-3605006-supplementary.pdf]

Appendix SA - Selected guides to predict central venous pressure based on inferior vena cava diameters and the inferior vena cava collapsibility index

The American Society of Echocardiography has suggested[12,13] that a combination of the widest (end-expiratory) inferior vena cava (IVC) diameter ≤2.1 cm and the inferior vena cava collapsibility index (IVCCI) >50% is indicative of central venous pressure (CVP) of 0-5 mmHg; a combination of a diameter >2.1 cm and an IVCCI >50% is indicative of CVP 5-10 mmHg; and a diameter >2.1 cm combined with an IVCCI <50% is indicative of CVP 10-20 mmHg. A more detailed classification of CVP was first introduced in 1990, incorporating a finer-graded combination of the widest IVC diameter and the IVCCI [14]. It was further refined in a 2015 literature review [15], as summarized in Table A1. In the present study, prediction of CVP was based on the criteria presented in Table A1.

Table S1. Relationship between the widest inferior vena cava (IVC) diameter, inferior vena cava collapsibility index (IVCCI), and corresponding (estimated) central venous pressure (CVP)[14].

Widest IVC diameter (cm)	Collapsibility Index (%)	Estimated CVP (mmHg)
< 1.5	100	0-5
1.5 – 2.5	> 50	6-10
1.5 – 2.5	< 50	11-15
> 2.5	< 50	16-20
> 2.5	0	> 20

Appendix SB - Methods to correct inferior vena cava diameters measured at increased levels of the intra-abdominal pressure

For both the end-inspiratory and the end-expiratory inferior vena cava (IVC) diameters, differences between the values at the intra-abdominal pressure (IAP) of 4-10 mmHg (“reference” level), and each of the 4 levels corresponding to intra-abdominal hypertension [11-15, 16-20, 21-25 and >25 mmHg (to 35 mmHg in the present patient sample)] were estimated with 95%CI, with adjustment for age, sex, and the use of mechanical ventilation. Since the IAP levels were defined in 5 mmHg increments, the generated estimates were used to correct (increase) the actually measured diameters in the following way: the IVC diameters measured at the two lowest IAP values in the respective category were increased by the amount equal to the lower limit of the 95%CI around the estimate; the IVC diameter measured at the IAP value equal to the middle value of the respective IAP category was increased by the amount equal to the point-estimate; the IVC diameters measured at the two highest IAP values in the respective category were increased by the amount equal to the upper limit of the 95%CI around the estimate.

Appendix SC - Patients with spontaneously increased intra-abdominal pressure and ascites: descriptive hemodynamics data

Table S2. Hemodynamics indicators. Data are count (percent) or median (Q1, Q3; min, max).

	All	Mechanically ventilated	Breathe spontaneously
N	36	18	18
Age (years)	58 (47-66; 34-78)	57 (41-64; 34-69)	61 (48-68; 41-78)
Men	29 (80.6)	15 (83.3)	14 (77.8)
Post-baseline measurements			
1	36 (100)	18	18
2	36 (100)	18	18
3	36 (100)	18	18
4	35 (97.2)	18	17
5	35 (97.2)	18	17
6	31 (86.1)	16	15
7	28 (77.8)	15	13
8	23 (63.9)	12	11
9	17 (47.2)	9	8
10	13 (36.1)	7	6
11	5 (13.9)	2	3
12	2 (5.6)	1	1
13	1 (2.8)	1	0
IAP (mmHg)			
Baseline	22 (19-25; 13-25)	22.5 (18.8-25.0; 14-35)	21.5 (18.8-26.5; 13-33)
After ascites removal	13 (10.2-16.5; 6-29)	12.5 (11.5-15.0; 6-28)	13.5 (10-18.2; 8-29)
Volume of ascites removed (L)	4.0 (2.9-4.9; 1.2-9.0)	3.7 (2.8-4.0; 1.8-6.0)	4.0 (3.2-6.1; 1.2-9.0)
CVP (mmHg)			
Baseline	14.5 (11-16; 5-20)	14.5 (11.8-15.2; 6-19)	13.5 (10.2-17.0; 5-20)
After ascites removal	13.0 (11-16; 5-20)	13.0 (11.8-16.0; 6-17)	12.5 (9.5-16.0; 5-20)
End-inspiratory IVC (cm)			
Baseline	0.9 (0.5-1.3; 0-2.2)	1.0 (0.5-1.2; 0.1-1.6)	0.9 (0.5-1.5; 0-2.2)
After ascites removal	1.5 (1.0-1.9; 0.4-2.6)	1.5 (1.2-1.8; 0.4-1.9)	1.8 (0.9-1.9; 0.6-2.6)
End-expiratory IVC (cm)			
Baseline	1.1 (0.7-1.5; 0-2.3)	1.1 (0.6-1.3; 0.1-1.7)	1.2 (0.8-1.5; 0-2.3)
After ascites removal	1.9 (1.6-2.1; 0.6-2.9)	1.8 (1.6-2.0; 0.6-2.6)	2.0 (1.6-2.4; 0.9-2.9)
IVCCI (%)			
Baseline	7.4 (2.7-22.4; 0-51.5)	7.0 (3.7-12.8; 0-42.0)	11.3 (2.2-36.7; 0-51.5)
After ascites removal	16.3 (8.9-38.5; 3.7-52.0)	14.9 (8.5-33.4; 4.8-50.0)	20.2 (8.8-43.6; 3.7-52.0)

CVP – central venous pressure, IAP – intraabdominal pressure, IVC – inferior vena cava, IVCCI – inferior vena cava collapsibility index

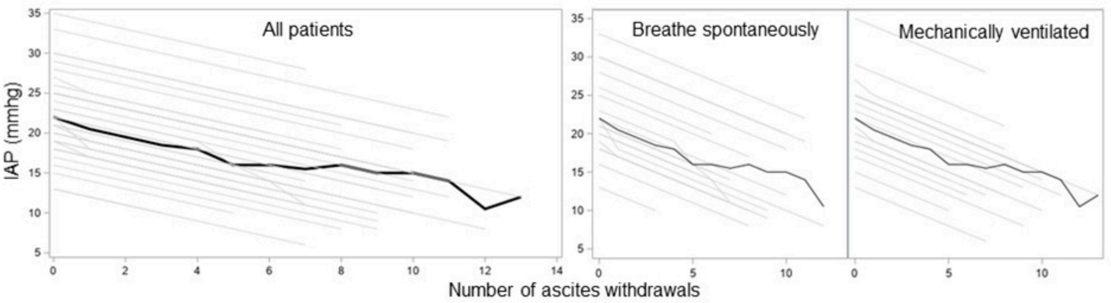

Figure S1. Development of intra-abdominal pressure (IAP) across the number of ascites withdrawals.

Appendix SD - Patients with normal intra-abdominal pressure at baseline and intra-abdominal pressure induction

Table S3. Effect of implementation of abdominal belt on intra-abdominal pressure (IAP) in patients with normal baseline IAP. Data are count (percent) or median (Q1, Q3; min, max).

	All	Mechanically ventilated	Breathe spontaneously
N	30	10	20
Age (years)	72 (61-76; 21-86)	73 (65-77; 52-86)	71 (58-75; 21-83)
Men	17 (56.7)	7 (70.0)	10 (50.0)
Baseline IAP (mmHg)	9.0 (7.7-10; 4.0-12.0)	8.0 (7.0-10.5; 5.0-12.0)	10.0 (8.0-10.0; 4.0-12.0)
Induced IAP (mmHg)			
Increased by 5	14.0 (12.7-15.0; 9.0-17.0)	13.0 (12.0-15.5; 10.0-17.0)	15.0 (13.0-15.0; 9.0-17.0)
Increased by 10	19.0 (17.7-20.0; 15.0-22.0)	18.0 (17.0-20.5; 15.0-22.0)	20.0 (18.0-20.0; 14.0-22.0)
Increased by 15	24.0 (22.7-25.0; 19.0-27.0)	23.0 (22.0-25.5; 20.0-27.0)	25.0 (23.0-25.0; 19.0-27.0)

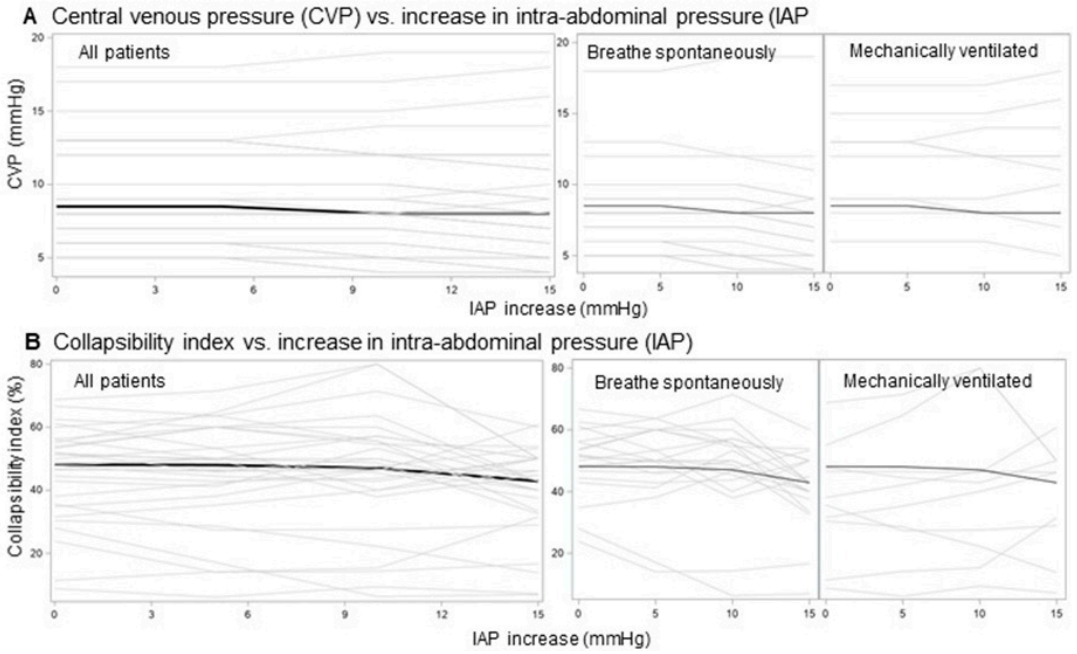

Figure S2. Effect of increasing intra-abdominal pressure (IAP) on central venous pressure (CVP) **(A)** and the inferior vena cava collapsibility index (IVCCI) **(B)**. Increase in IAP induced no consistent changes in CVP or IVCCI. Only the IAP increase of 15 mmHg resulted in minor (adjusted) reductions vs. baseline in CVP [CVP had to be log-transformed, and the estimated reduction was -10.0% (95%CI -11.7 to -6.2)], and CI [-5.1% (95%CI -8.6 to -1.6)].

Appendix SE - Correction of inferior vena cava diameters measured at increased intra-abdominal pressure

Table S4. Data used to estimate difference in measured end-inspiratory and end-expiratory inferior vena cava (IVC) diameters between those taken with intra-abdominal pressure (IAP) in the range between 4 and 10 mmHg (reference range), and those taken at higher values of IAP (11-15, 16-20, 21-25 or 26-35 mmHg). Shown are numbers of evaluated patients and values taken at each IAP level and mean (95%CI) IVC diameters at the respective IAP level, adjusted for age, sex, and mechanical ventilation.

IAP (mmHg)	N subjects/values	End-inspiratory (95%CI)	End-expiratory (95%CI)
4-10 (reference)	36/48	1.37 (1.12-1.52)	2.04 (1.88-2.20)
11-15	60/119	1.22 (1.08-1.36)	1.74 (1-58-1.89)
16-20	60/142	0.99 (0.84-1.14)	1.39 (1.24-1.54)
21-25	48/104	0.80 (0.66-0.95)	1.10 (0.95-1.26)
26-35	14/41	0.56 (0.40-0.71)	0.72 (0.55-0.99)

Table S5. Adjusted mean differences (95%CI) between the end-inspiratory or end-expiratory inferior vena cava diameters taken at normal intra-abdominal pressure (4-10 mmHg, reference), and at higher levels of IAP estimated based on data in Table S1.

IAP (mmHg)	End-inspiratory (95%CI)	End-expiratory (95%CI)
4-10 (reference)	0	0
11-15	-0.15 (-0.20, -0.10)	-0.31 (-0.39, -0.24)
16-20	-0.38 (-0.42, -0.32)	-0.65 (-0.72, -0.58)
21-25	-0.57 (-0.63, -0.51)	-0.94 (-1.02, -0.86)
26-35	-0.82 (-0.90, -0.73)	-1.33 (-1.44, -1.21)
